# Redescription of the male of *Ixodes festai* Rondelli, 1926 (Ixodida: Ixodidae) on specimens from Sardinia (Italy)

**DOI:** 10.1051/parasite/2011183235

**Published:** 2011-08-15

**Authors:** C. Contini, C. Palmas, V. Seu, L. Stancampiano, F. Usai

**Affiliations:** 1 Dipartimento di Scienze della Vita e dell’Ambiente, Macrosezione Biomedica, Università di Cagliari Italy; 2 Dipartimento di Scienze Mediche Veterinarie, Università di Bologna Italy

**Keywords:** Ixodidae, *Ixodes festai*, description, male, Sardinia, Ixodidae, *Ixodes festai*, description, mâle, Sardaigne

## Abstract

*Ixodes festai* Rondelli, 1926 is a poorly known bird parasite tick. Its immature forms have not been described yet, while the adult forms only insufficiently, especially the male. In this note the presence of the male of *Ixodes festai* for the first time in Sardinia (Italy) is reported and a detailed redescription is provided. Morphometric data as well as photographs performed both with optical and electron microscope (ESEM FEI Quanta 200) are also shown.

## Abstract (fr)

*Ixodes festai* Rondelli, 1926 est une espèce mal connue, parasite des oiseaux. Les stades immatures sont toujours actuellement non décrits, et les stades adultes eux-mêmes restent encore très partiellement décrits, notamment le mâle. Dans cet article, les auteurs décrivent le mâle de l’Ixodidae *Ixodes festai* signalé pour la première fois en Sardaigne (Italie), et une nouvelle description détaillée est fournie. En outre, des données morphométriques et des photographies prises par un microscope optique et par un microscope électronique ESEM FEI Quanta 200 complètent cet article.

The Ixodidae *Ixodes festai* was originally described by Rondelli in 1926 on a female specimen collected in Derna (Libya) from *Alectoris barbara* (Phasianidae).

Subsequently, Arthur erroneously ascribed to the species *Ixodes festai* specimens collected in the forest of Néfifik (Morocco) on *Oryctolagus cuniculus* (Leporidae) providing a redescription of the female and the first description of the male (Arthur, 1957, 1961, 1965). Indeed, Arthur confused *I. festai* with *Ixodes ventalloi* Gil Collado, 1936 which is a tick parasite of rabbits at every development stages, rarely found on ground dwelling birds.

Since then, the two *Ixodes* species were described, confused and described again until 1978, when they have been definitively split by Gilot and Perez, that pointed out the error into which Arthur fell. *I. festai* is a bird parasite tick whose main hosts are *Alectoris barbara* (Phasianidae), *Phasianus colchicus* (Phasianiade) and Turdiade, mostly *Turdus* sp. This ectoparasite mainly occurs in the West Mediterranean, where it is known in Tunisia, Morocco, Libya, France (including Corsica) (Pérez-Eid, 2007). Its presence has also been recorded in Poland (Siuda *et al.*, 1991; Siuda *et al.*, 2006) and in Switzerland (Papadopulos *et al.*, 2001).

Collected samples have always been few in number, confirming the rarity of the species. The male of *I. festai* remained moreover unknown until 2007, when Pérez-Eid described the male among specimens collected by Gilot in Bormes-les-Mimosas, Var, France (Pérez-Eid, 2007).

The presence in Italy of *I. festai* was reported for the first time by Contini (1998) who collected 24 individuals, all females, parasitizing mainly *Turdus philomelos* but also *Turdus merula* (Turdidae). The sampling was performed in the countryside of the Teulada and Capoterra municipalities (Cagliari province, Sardinia). More recently Iori *et al*. (2004) reported the presence of *I. festai* in two Italian islands, Ventotene (Latina province, Lazio) and Montecristo (Livorno province, Tuscany), examining two unidentified ingorged females collected by Manilla in 1990 on *Turdus torquatus* and *T. philomelos* (Turdiadae). This record, which the authors stated to be the first in Italy, did not take into account the previous report of Contini (1998).

To date, *I. festai* remains a poorly known species. Its immature forms have not been described yet, while the adult forms only insufficiently. In particular the male has only been barely described by Pérez-Eid (2007). The description given by this latter, indeed, is very brief, the drawings are very schematic and photographs of the various anatomical details are not present.

In addition the male of *I. festai* has never been reported in Sardinia (Italy) before. In this paper we provide a detailed description of the male of *I. festai*, thus deepening the one given by Pérez-Eid (2007).

## Materials and Methods

Following the publication of Contini (1998), we organized a second research in February 2000, always in the same locations, which allowed the capture of over one hundred specimens of *I. festai*, all of them collected from *T. philomelos* and *T. merula*.

Examining the collected material, five males and five females in copula were detected, as well as a free male lying on the abdomen of a female. *I. festai* specimens were mainly associated with *T. philomelos* in rural areas of the municipalities of Teulada (Monte Perdosu, Monte Nappa and Monte Sebera, Cagliari province) and Capoterra (San Gregorio and Poggio dei Pini, Cagliari province).

The specimens were first treated in heated 10% KOH solution for 20 minutes, washed several times in a mild solution of acetic acid and distilled water, then in distilled water only and finally mounted in Faure’s liquid. The morphological characters were studied in the five males in copula. The morphometric data are presented in Table I, where minimum, average and maximum values are expressed in millimeters. Measures were taken with a micrometer slide.

**Table I.** Morphometric data of male of *Ixodes festai* (4 specimens measured).

		Dimensions (mm) or ratio		
	Measure N	Mean	Min	Max
Length of idiosome along the median longitudinal axis	1	1.3	1.2	1.4
Max width at peritremes	2	0.9	0.9	0.9
Length of scutum along the median longitudinal axis	3	1.2	1.2	1.3
Max width of dorsal scutum	4	0.7	0.7	0.7
Distance between scapulae	5	0.3	0.3	0.3
Max length of basis capituli along the median longitudinal axis	6	0.2	0.2	0.2
Max length of basis capituli at cornua	7	0.2	0.2	0.2
Distance between cornua	8	0.2	0.2	0.2
Max length of hypostome	9	0.2	0.2	0.2
Max width of hypostome	10	0.1	0.1	0.1
Max length of palp segment II along the median axis	11	0.1	0.1	0.1
Max length of palp article III along the median axis	12	0.2	0.1	0.2
Max width of palps	13	0.1	0.1	0.1
Ratio of width of palp to length of its II segment		1:1	1:0.9	1:1.1
Ratio of width of palp to length of its III segment		1:1.2	1:1.2	1:1.2
Length of tarsus I	16	0.4	0.4	0.4
Width of tarsus I	17	0.1	0.1	0.1
Width to length ratio of tarsus I		1:3.7	1:3.5	1:4.1
Longitudinal diameter of ring of anal valve	19	0.1	0.1	0.1
Longitudinal diameter of peritreme	20	0.2	0.2	0.2

SEM photographs were performed on specimens stored in 70% ethanol solution, using the ESEM FEI Quanta 200 microscope.

## Description of the Male of *I. Festai*

**Fig. 1.**
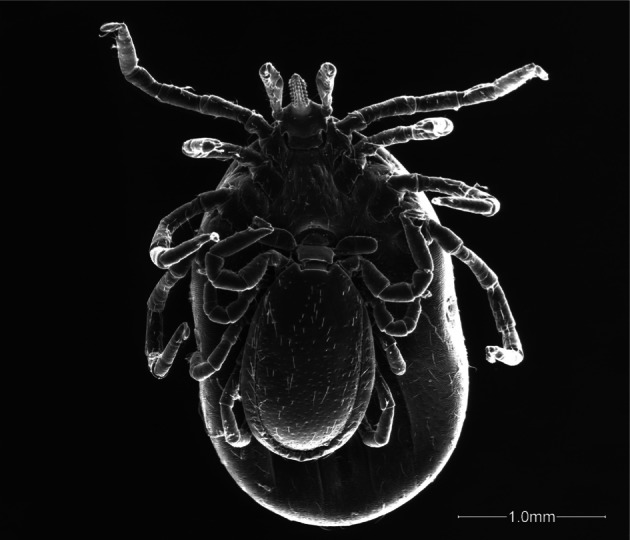
Male and female of *Ixodes festai* mating.

**Fig. 2.**
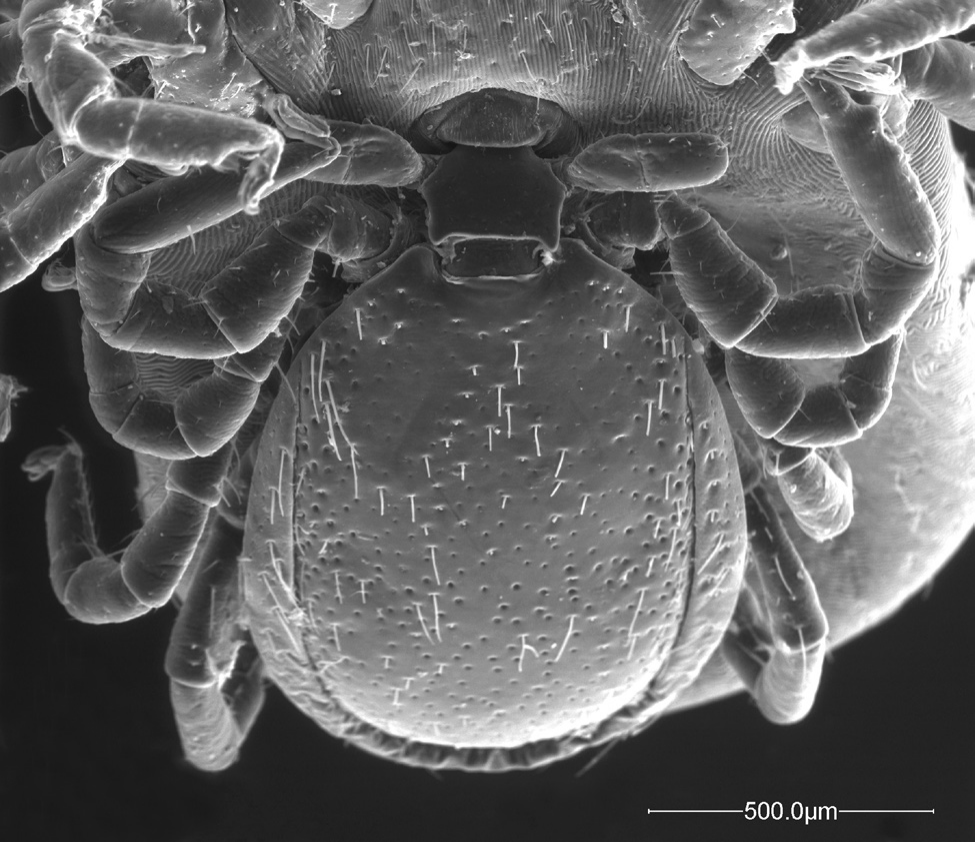
Particular of the male in figure 1.

**Fig. 3.**
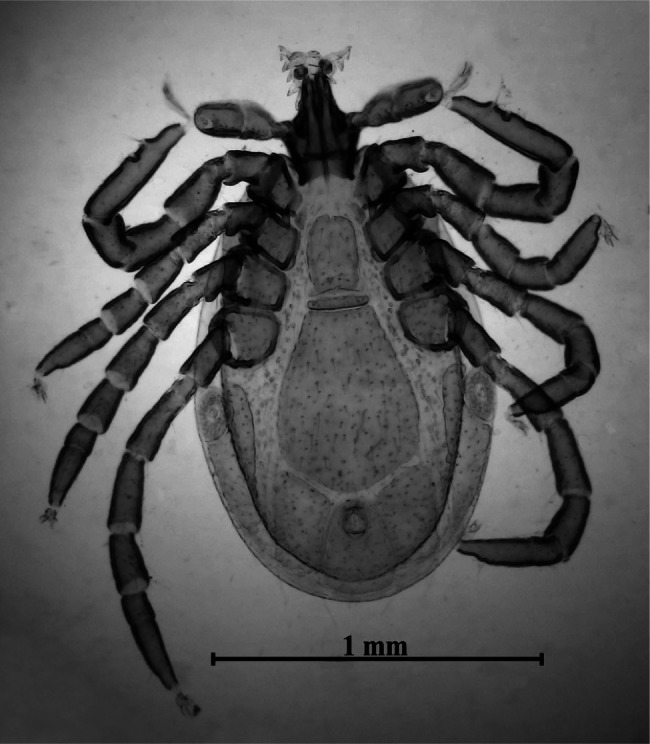
Male of *Ixodes festai* clarified in KOH 10% solution and mounted in Faure’s liquid, lying dorsally.

Body very small, average length 1.7 mm (Table I, Figs 1, 3), of oval form, uniformly brown coloured, feet included. Dorsal shield covered with white rare bristles not uniformly distributed, absent in two small anterolateral areas, just below the scapulae (Figs 1, 2). Punctuations of a single type, large, distributed mainly in the posterior two thirds of the shield (Fig. 2). Lateral grooves deep and continuous, interrupted near the scapular areas. Cervical grooves very little marked (Fig. 2). Basis capituli plate (Fig. 2), roughly as long as wide in the length and width; posterior margin almost straight and with two robust triangular cornuae with rounded apex.

**Fig. 4.**
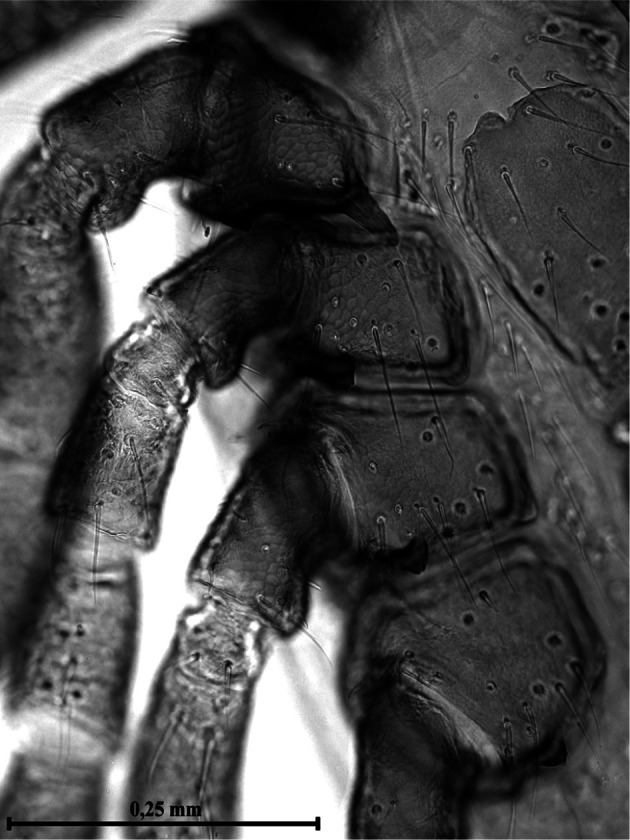
Coxae I-IV of the male of *Ixodes festai*.

**Fig. 5.**
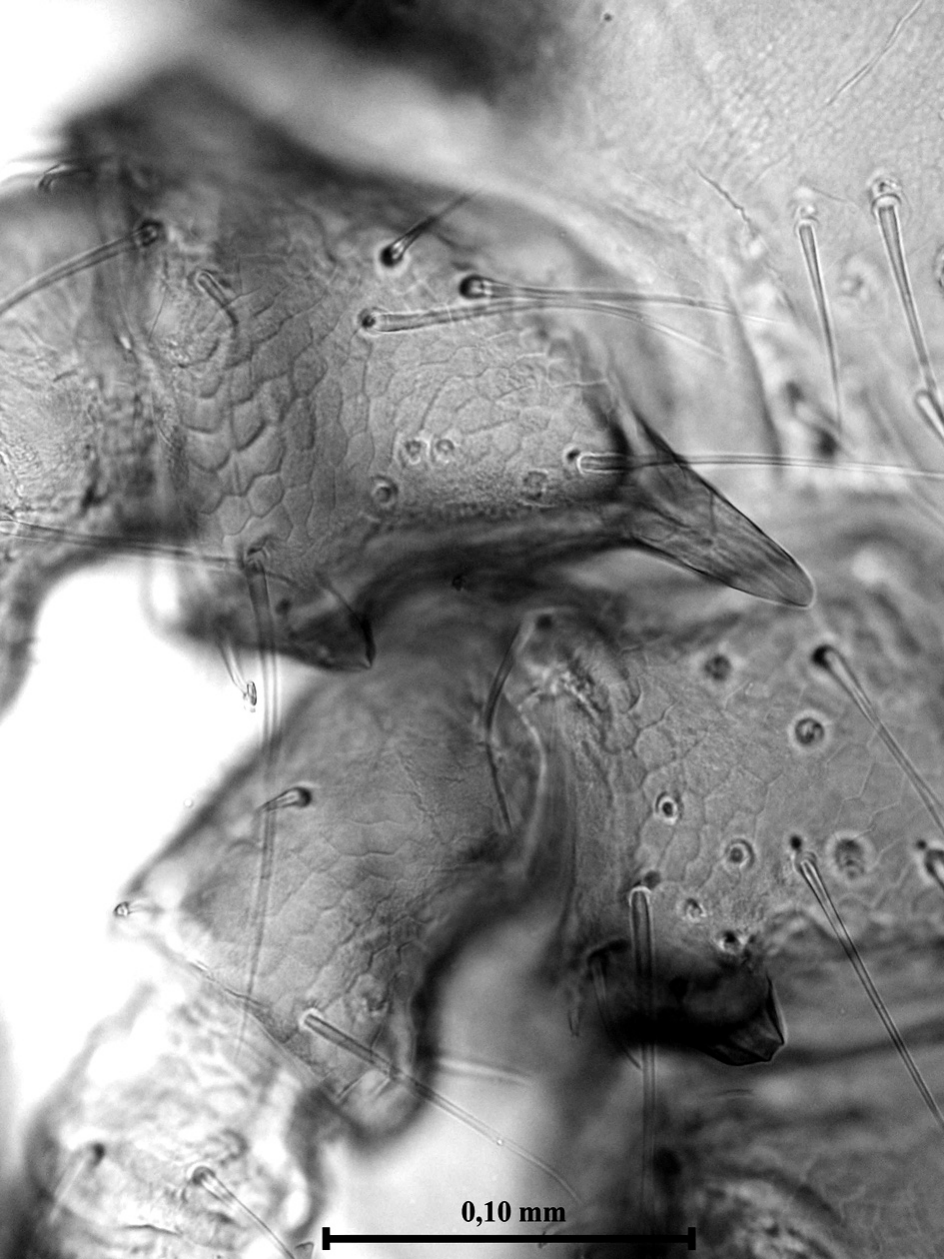
Particular of figure 4: internal and external thorn of the coxa I and external thorn of the coxa II of the male of *Ixodes festai*. The terminal cusps of the external thorns are well visible.

**Fig. 6.**
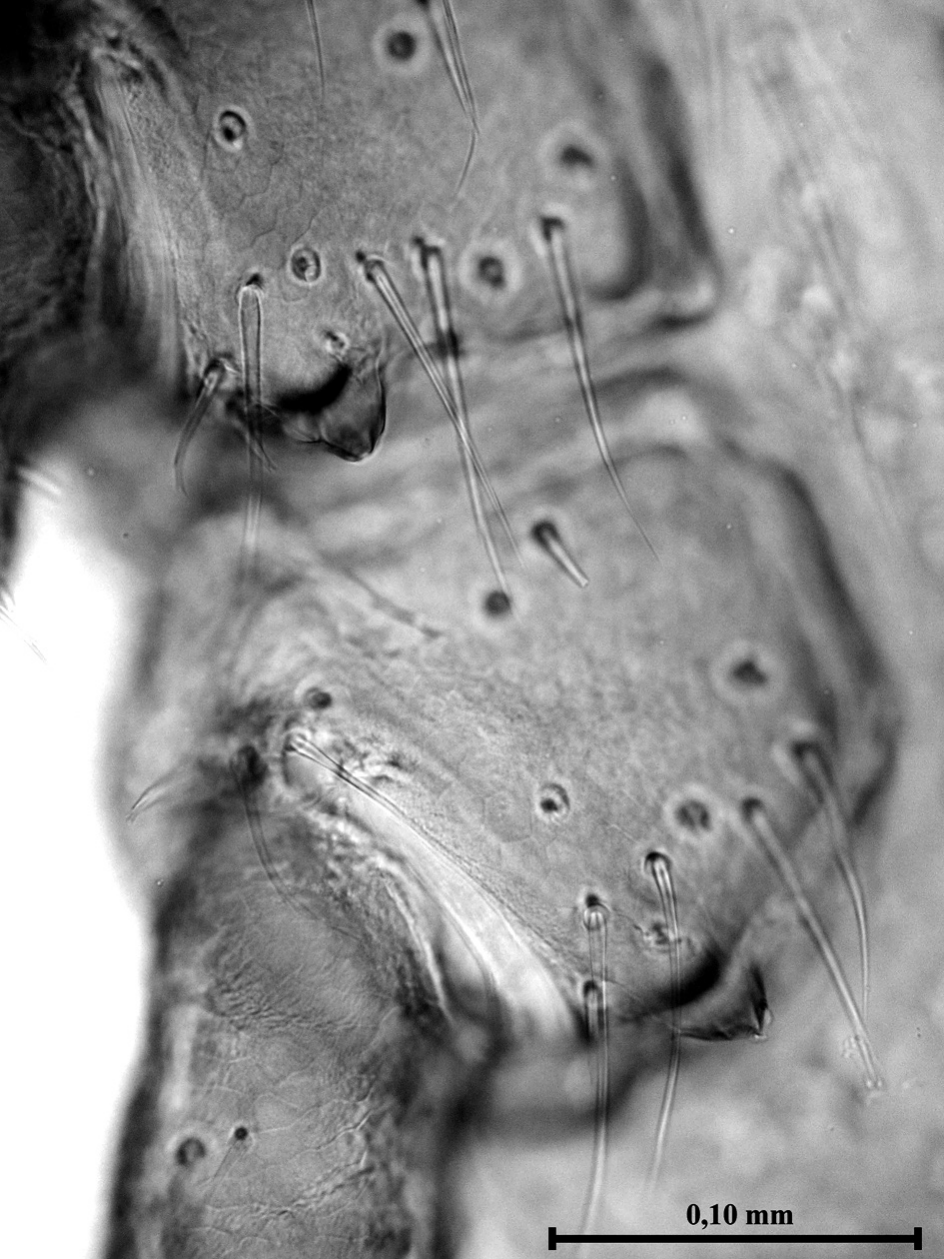
Particular of figure 4: external thorns of the coxae III and IV of the male of *Ixodes festai.* The terminal cusps of the external thorns are well visible.

**Fig. 7.**
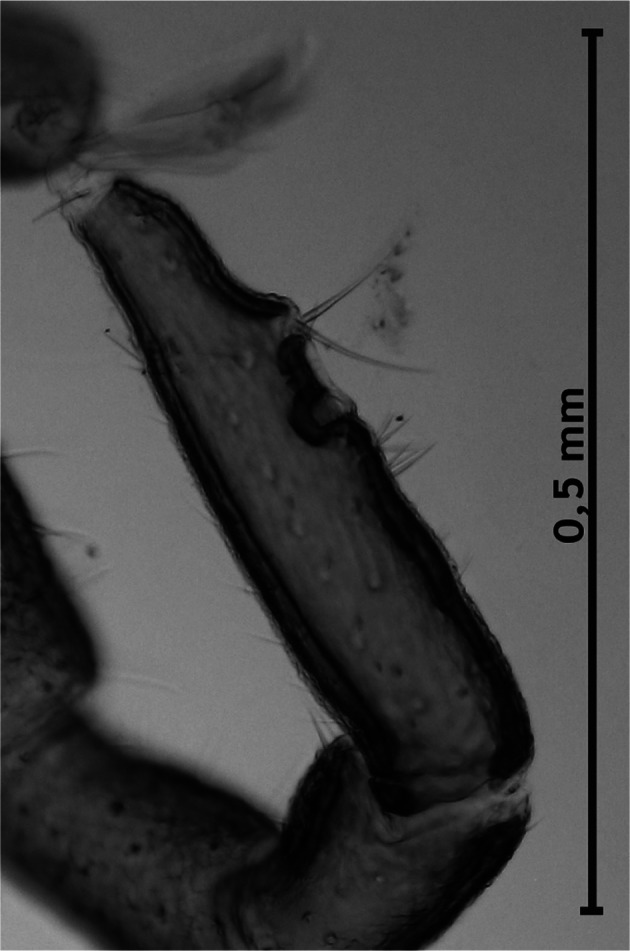
Tarsus of the leg I.

Dorsal surface of the basis capituli with 12-13 punctuations. The ventral surface is equipped with seven shields (Fig. 3) not very dissimilar in form and disposition from those of other species of Ixodinae. Coxae (Fig. 4) with long bristles. Coxa I (Fig. 5) provided with robust internal spur, straight, blunt, long about twice the width of the base and slightly placed on the coxa II; coxae I-IV (Figs 4-6) bearing external thorns whose ends are provided of 1-3 small cusps; coxa IV with, occasionally, a second rudimentary external thorn (Fig. 6); anterior tarsi as in Fig. 7. Spiracles rounded, just smaller than coxae IV (Fig. 3).

**Fig. 8.**
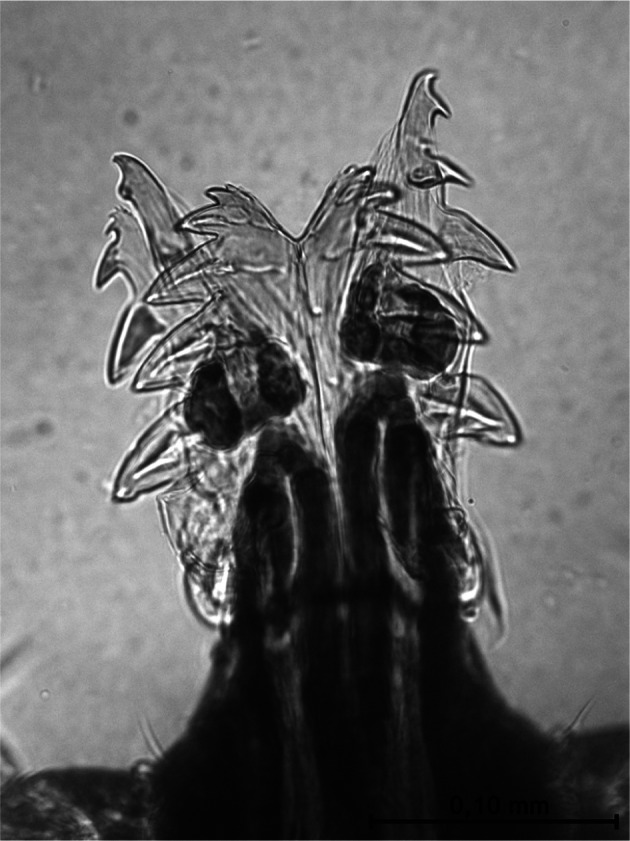
Hypostome of the male of *Ixodes festai*.

Hypostome (Fig. 8) short, provided with small teeth, with apex hollow V-shaped. Six sharp teeth are also present on each side which increase progressively in size from apex to base. The median region includes five rows on each side of rudimentary teeth similar to small crenations. The first row of teeth, just below the apex, consists of ovoidal elements.

## Discussion

The new samples gathered in Sardinia are interesting as they provide further information. In particular they confirm the persistency of *I. festai* within the same biotope, as remarked by Contini (1998), which is an interesting aspect for migratory bird parasites. Furthermore this report considerably enlarge the amount of known samples, and does not confirm the conclusion drawn by Pérez-Eid regarding the sporadicity of this species on the basis of the small number of individuals in most collections.

The samples also confirm *Turdus* sp. as key host species in particular in Italy, which is an important aspect for the biology of this ectoparasites until now only rarely detected.

Below, we provide the main morphological characters useful for differentiating the male of *I. festai* from other Italian Ixodinae.

According to Manilla (1998) the Italian fauna of the genus *Ixodes* Latreille includes the following four species: *I. ricinus* (Linnaeus, 1758), *I. gibbosus* Nuttal, 1916, *I. acuminatus* Neumann, 1901 and *I. ventalloi* Gil Collado, 1936. Males of *I. festai* are separable from males of these species using the following diagnostic characters:

- cornuae present in *I. festai*, *I. ventalloi* and *I. acuminatus*, absent in *I. ricinus* and *I. gibbosus*;
- auriculae strongly reduced in *I. festai* and *I. ventalloi*, absent in *I. acuminatus*, present and forming a right angle in *I. ricinus*.
- internal thorn of the coxa I robust, straight and blunted in *I. festai*, pointed and arcuated in *I. ventalloi*;
- tarsus I long and slender in *I. festai*, shorter, wider with untapered end in *I. ventalloi*;
- basis capituli with margins subparallel dorsally in *I. festai*, significantly divergent anteriorly in *I. acuminatus*;
- internal thorns of the coxae II and III rudimentary in *I. festai*, more evident in *I. acuminatus*.

In conclusion, this work besides providing a detailed description of the male of *I. festai*, adds new information regarding the distribution of this species. Further considerations about the reasons for such a strong presence of *I. festai* in Sardinia, would need to be deepened by further research.
